# Pandemic Fatigue and Preferences for COVID-19 Public Health and Social Measures in China: Nationwide Discrete Choice Experiment

**DOI:** 10.2196/45840

**Published:** 2024-06-27

**Authors:** Meng Yang, Zonglin He, Yin Zhang, Taoran Liu, Wai-kit Ming

**Affiliations:** 1 School of Medicine Jinan University Guangzhou China; 2 Division of Life Science Hong Kong University of Science and Technology Hong Kong China (Hong Kong); 3 Department of Medicine The University of Hong Kong Hong Kong China (Hong Kong); 4 Department of Infectious Diseases and Public Health City University of Hong Kong Hong Kong China (Hong Kong)

**Keywords:** pandemic fatigue, preference, public health and social measures, discrete choice experiment, COVID-19

## Abstract

**Background:**

Information on the public’s preferences for current public health and social measures (PHSMs) and people’s mental health under PHSMs is insufficient.

**Objective:**

This study aimed to quantify the public’s preferences for varied PHSMs and measure the level of pandemic fatigue in the COVID-19 normalization stage in China.

**Methods:**

A nationwide cross-sectional study with a discrete choice experiment and psychometric scales was conducted to assess public preferences for and attitudes toward PHSMs, using the quota sampling method. The COVID-19 Pandemic Fatigue Scale (CPFS) was used to screen fatigue levels among respondents. The multinomial logit model, latent class model, and Mann-Whitney test were used for statistical analysis. We also conducted subgroup analysis based on sex, age, monthly income, mental health status, and pandemic fatigue status.

**Results:**

A total of 689 respondents across China completed the survey. The discrete choice experiment revealed that respondents attached the greatest importance to the risk of COVID-19 infection within 3 months (45.53%), followed by loss of income within 3 months (30.69%). Vulnerable populations (low-income populations and elderly people) were more sensitive to the risk of infection, while younger respondents were more sensitive to income loss and preferred nonsuspension of social places and transportation. Migrants and those with pandemic fatigue had less acceptance of the mandatory booster vaccination and suspension of transportation. Additionally, a higher pandemic fatigue level was observed in female respondents, younger respondents, migrants, and relatively lower-income respondents (CPFS correlation with age: *r*=–0.274, *P*<.001; correlation with monthly income: *r*=–0.25, *P*<.001). Mandatory booster COVID-19 vaccination was also not preferred by respondents with a higher level of pandemic fatigue, while universal COVID-19 booster vaccination was preferred by respondents with a lower level of pandemic fatigue.

**Conclusions:**

Pandemic fatigue is widely prevalent in respondents across China, and respondents desired the resumption of normal social life while being confronted with the fear of COVID-19 infection in the normalization stage of COVID-19 in China. During future pandemics, the mental burden and adherence of residents should be considered for the proper implementation of PHSMs.

## Introduction

The transmission of the SARS-CoV-2 Omicron variant led to a sharp rise in infected cases in mainland China, spreading from major cities like Guangzhou and Shanghai to the entire country [1]. To contain the transmission of the virus, various public health and social measures (PHSMs) have been adopted at municipal and provincial levels in China under the dynamic zero-COVID policy [2]. These measures include suspension of public transport, closure of public places, closed-off community management, mandatory nucleic acid testing, home quarantine, and isolation of infected and suspected cases, among others [3-5]. However, the severity of the epidemic combined with high-level public health policies during the Omicron wave had significant impacts on the normal life of citizens from diﬀerent dimensions and may have resulted in mental health issues [6].

Fatigue issues have been noticeable during the COVID-19 pandemic, especially the adverse psychological impacts of nonpharmacological interventions (NPIs) [7]. In China, the prevalences of anxiety and depression symptoms were reported to be 29% and 37.1%, respectively, during the COVID-19 pandemic in 2020 [8]. A study in Italy found that 38% of the general population had psychological distress during the early stage of the COVID-19 pandemic [9]. Among them, vulnerable populations, including elderly people [10,11], migrant workers [12,13], children [14-16], adolescents [16,17], and individuals with pre-existing mental illness [18,19], may have a greater risk of psychological diﬃculties due to increased exposure to external adverse circumstances. The prevalence of mental disorders was found to be higher during the Omicron wave than during the wild-type wave. For example, the study by Lu et al [20] found that among nonmedical and medical staff, the prevalence rates of anxiety were 55.0% and 47.3%, respectively, and the rates of depression were 62.4% and 53.4%, respectively.

Long-term COVID-19 public health policy may result in pandemic fatigue [21,22], causing a decline in public compliance [23,24]. Changes in people’s perceptions of risk assessment have also led to behavioral changes [23,25]. Rayani et al [26] reported that higher levels of risk perception might allow people to maintain positive preventive behaviors. A study by Alijanzadeh et al [27] in Iran also showed that the risk perception of individuals can inﬂuence preventive COVID-19 behaviors through their fear of COVID-19 and trust in the health care system. Meanwhile, public participation at the policy level in preventive behavior, disease response, and surveillance has become increasingly important [28]. Information on public perceptions and attitudes toward social distancing measures is prominent in the unofficial media. In contrast, formal research evidence on the public’s preferences toward the current PHSMs and people’s mental health problems during the Omicron wave under the strengthened COVID-19 policy has been insuﬃcient. Moreover, no studies to date have captured the desirability of the different PHSMs toward the pandemic in China or have captured the general public’s willingness to trade. Such insufficient information on the general public’s pandemic fatigue and preference may hinder priority settings when no single PHSM can sufficiently combat the transmission of the virus.

In the context of PHSMs, this study aimed to explore the public’s preferences and preference homogeneities and heterogeneities for varied PHSMs. Furthermore, based on an assessment of the current level of prevention and control measures in participants’ regions, this study considered the impact of PHSM fatigue on preferences according to the epidemic fatigue scale [29,30].

## Methods

### Overview

In this study, we used various instruments to investigate mental health problems among the general population, especially migrant workers and those who work in nonregistered locations for 3 months or more [31]. The ﬁrst instrument was a discrete choice experiment (DCE) questionnaire, which had a survey-based experiment design that solicited and quantified respondents’ utilities and preferences toward a set of attributes and levels of PHSMs. Following the DCE questionnaire, a Likert psychometric scale of pandemic fatigue was used to measure the respondents’ perceptions of the current PHSMs and levels of pandemic fatigue. Additionally, we conducted a subgroup analysis [32] to explore the heterogeneities based on demographic information and socioeconomic status, and a comparison was conducted of the preferences of respondents with relatively low pandemic fatigue levels and those with high fatigue levels.

### Respondents

The inclusion criteria of this study were age of at least 18 years and absence of cognitive impairments (self-report). Respondents were recruited and selected through an online social media advertising platform (Credamo Inc), which has over 3 million samples and covers all provincial administrative regions in China [33-36]. Credamo randomly distributed the survey in 31 provinces of China (excluding Hong Kong, Macao, and Taiwan). Specifically, we provided a quota size of 350 per sex group and 140 per age group (oversampled). According to this census, the population of China was approximately 1.411 billion, with 51.24% male individuals (723.34 million) and 48.76% female individuals (688.44 million) [37,38], leading to a sex ratio of approximately 105.07 male individuals for every 100 female individuals. We also referred to the National Bureau of Statistics of China [37] for age-specific quota design, with 63.35% in the age group of 15 to 59 years and 18.70% in the age group of 60 years or older. However, due to budget restrictions and practical issues during the pandemic, we considered a 1:1 ratio per sex group and 140 individuals per age group for the data collection platform. No personally identiﬁable information was collected as the survey was anonymous. Consent was obtained when the respondents actively pressed the button marked “I have been informed with suﬃcient information of the study and agree to participate in this study” after viewing the introductory section of the questionnaire where the background and objectives of the study were presented. Respondents could only access the questionnaire if they consented and reported that they were 18 years or older and did not have cognitive impairments. The translation of the original survey has been provided in Multimedia Appendix 1. Respondents received RMB 20 (US $2.76) as an incentive for participation.

### Data Collection

An anonymous self-administered survey created using Lighthouse Studio (version 9.9.1; Sawtooth Software) was distributed from July 01, 2022, to September 30, 2022, and respondents from the entire country were considered for inclusion. The minimum sample size requirement of this study was calculated using the rule of thumb approach proposed previously [39]. Speciﬁcally, the equation for sample size calculation was as follows:

N > 500c / (t × a)

where t refers to the choice tasks in the survey, a refers to the number of alternatives, and c refers to the number of analysis cells. Speciﬁcally, the number of analysis cells c in this study refers to the largest number of levels for any of the attributes. As such, the minimum sample size in this study should be 125 respondents. Moreover, according to the standard parametric approach [40] of sample size calculation, the minimum sample size is 267 (Multimedia Appendix 2).

All the questions were close-ended, with tick boxes provided for responses and no question skipping allowed. No data were stored if the questionnaire website was closed before the completion of the survey.

### Survey and DCE Design

The survey of this study had 4 main sections. Speciﬁcally, in the ﬁrst section, we aimed to solicit respondents’ demographic information, including socioeconomic information (age, sex, education level, religion, marital status, occupation, income level, current residence, and registered permanent residence city).

In the second section of the survey, respondents’ vaccination history and medical history were collected. Respondents were asked how many doses of COVID-19 vaccination they have received, whether they have ever been diagnosed with or are currently experiencing psychological diseases (eg, depression, anxiety, obsessive-compulsive disorder, phobia, bipolar disorder, neurasthenia, schizophrenia, and personality disorder), and whether they have ever been infected with COVID-19. If respondents answered that they had been diagnosed with or are currently experiencing psychological diseases, they were required to answer what speciﬁc disease they encountered, the severity of the disease, and whether they have ever received or are currently receiving treatment. In addition, if respondents indicated that they had been infected with COVID-19, they were required to provide information about how they found out that they had been infected, their symptoms and complications, and their date of hospitalization and discharge.

The third section of the survey was the DCE. Respondents were presented with 9 sets of scenarios, and in each scenario, respondents faced 3 hypothetical responses, namely, “option A,” “option B,” and “neither.” Respondents were required to select the measure that they felt most satisﬁed with. The attributes and levels of diﬀerent measures of the DCE were determined by a literature review [32,41,42] and consultation with local epidemiologists and experts, and according to design guidelines for DCE [43]. As a result, we determined 8 attributes in our study: (1) Risk of COVID-19 infection within 3 months; (2) Closure of social occasions; (3) Suspension of on-campus educational activities; (4) Suspension of public transportation; (5) Contact tracing, isolation, and quarantine; (6) Nucleic acid screening program; (7) Mandatory booster vaccination; and (8) Loss of income in 3 months. All the attributes and levels selected in the study have been summarized in Table 1.

**Table 1:** Attributes and levels selected in the discrete choice experiment survey.

Attributes	Levels
Risk of COVID-19 infection within 3 months	0% 20% 40% 60% 80% 100%
Closure of social occasions	Yes No
Suspension of on-campus educational activities	Yes No
Suspension of public transportation	Full suspension Suspension in high-risk areas Normal operation
Contact tracing, isolation, and quarantine	Voluntary Compulsory None
Nucleic acid screening program	Only high-risk units, workplaces, and vulnerable public Nucleic acid screening for all None
Mandatory booster vaccination	Universal vaccination Only high-risk groups are vaccinated (long-term patients, people over 60 years old, etc) None
Loss of income in 3 months	0% 20% 40% 60% 80% 100%

**Figure 1:**
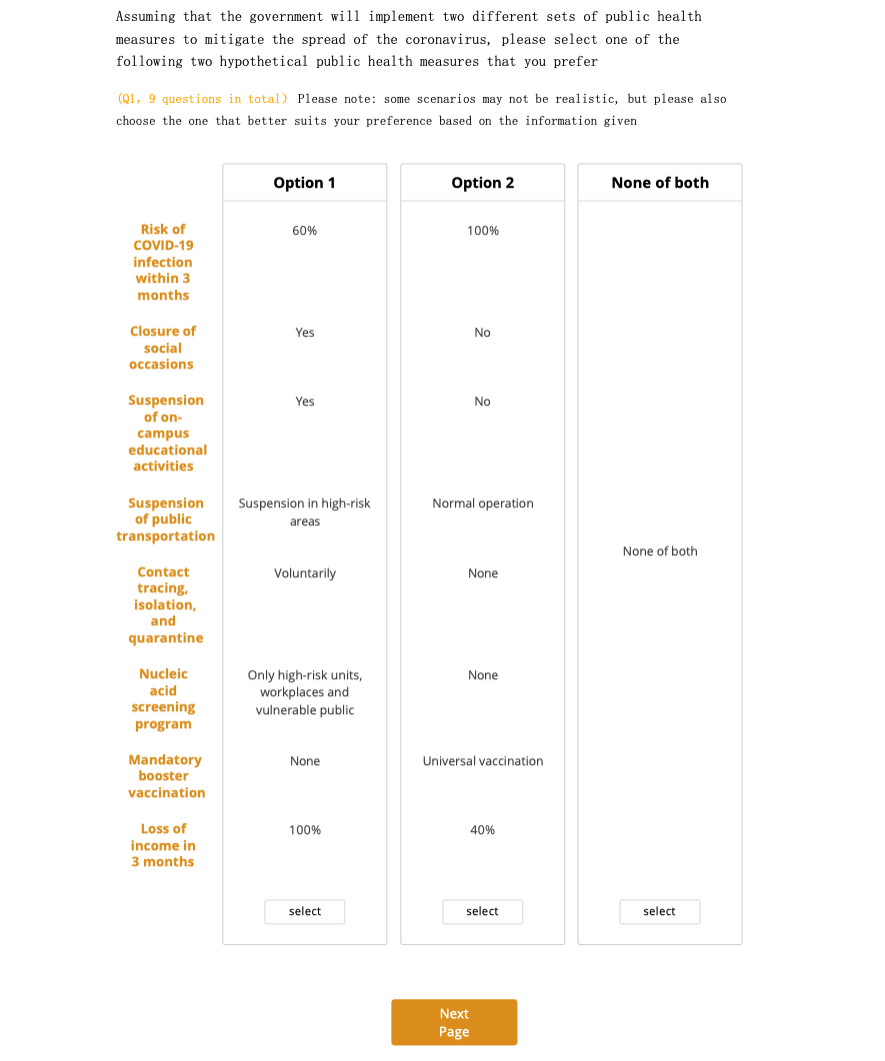
A sample of a hypothetical choice task in the discrete choice experiment (DCE) survey. Nine task choices in total were present in the DCE part. Each task choice contained 2 options (option 1 and option 2) and an “opt-out” option (none of both). Options were characterized by 8 attributes and random levels. Respondents were required to select an option from among the 2 options and the “opt-out” option.

A sample of a hypothetical choice task is shown in Figure 1. The levels in task choices of diﬀerent versions were designed according to the principles of (1) orthogonality and (2) balance. The task choices in the DCE section were 8 random choices and 1 ﬁxed choice. We used the ﬁxed choice for further data quality control. Since the DCE questionnaire is relatively more complicated for respondents to understand and such cognitive burden imposed on respondents may lead to some bias in their selection, we added specific text and forced respondents to stay on the questionnaire page for at least 1 minute and carefully read the text to help them better understand what discrete choice tasks are and how to select the choices subsequently. Details are provided in Multimedia Appendix 1.

The description was as follows:

In this part, you will face a series of tasks; these are called discrete choice tasks, a method we use to understand preferences and decision-making processes. Each task will offer you two hypothetical options and a “none” option, each with a set of attributes or features. Your task is to choose the option that you prefer or that you would most likely choose in real life. Please read the descriptions of each option carefully. Each option is different, with its unique set of attributes or characteristics. Remember, there's no right or wrong answer here. We are interested in your genuine preferences. Choose the option that best aligns with what you would prefer in real life, based on the attributes presented. Some scenarios may not happen in real life since they were hypothetical; however, please also select an alternative based on your own preferences.

According to the full factorial design, there were 11,664 (6×2×2×3×3×3×3×6) policies and 11,664×11,663 task choices. To significantly reduce the complication of the design in order to ensure that respondents could complete the tasks, we applied a fractional factorial design based on balance (the frequencies of attribute levels are roughly equal across all tasks) and orthogonality (the frequencies of attribute pairs are roughly equal across the tasks) principles.

Psychological Likert scales were included in the fourth section of the survey, and pandemic fatigue was assessed on a 5-point Likert scale. The reliability (α=.885) and validity (Kaiser-Meyer-Olkin measure=0.737) of the Likert scale for pandemic fatigue were tested. The pandemic fatigue model designed by Lilleholt et al [29] was used to ask about demotivation toward COVID-19 PHSMs and the desire to know the development of the epidemic. We adjusted the pandemic fatigue model in our study. The adjusted pandemic fatigue model contained a series of questions on public attitudes or views on the strengthening of relevant measures for epidemic prevention and control at the current stage and a series of scales to measure the public’s pandemic fatigue. The first question asked about local confirmed cases in the respondent’s living area (town, county, and district), and it was followed by a question that asked about the current PHSMs in that area. The third question contained a scale on the respondent’s perceived risk of being infected with COVID-19. The fourth question contained a scale to measure the respondent’s perceptions of the current measures. Subsequently, the fifth question assessed the epidemic prevention fatigue situation under the current situation of strengthened epidemic prevention measures.

### Statistical Analysis

Descriptive analysis was performed to describe respondents’ demographic information and socioeconomic information, as well as information regarding migration, COVID-19 vaccination history, mental health disease history, exposure to COVID-19, infection with COVID-19, and experience of closed-off community management (entrance numbers were minimized, checking points were set up in communities, entry permits were limited, face mask wearing was required, health monitoring was enhanced, and only registered personnel and vehicles were allowed to pass through).

We used the multinomial logit (MNL) model to quantify respondents’ relative utilities among all respondents. The MNL model of this study followed the random utility maximization theory [44]. We calculated the odds ratio (OR) and 95% CI based on respondents’ relative utilities among levels and attributes to further measure respondents’ preferences. In addition, we applied the latent class model (LCM) to determine how respondents’ preferences diﬀered according to group membership. We used the Akaike Information Criterion (AIC) and Bayesian Information Criterion (BIC) to determine the appropriate number of groups among the respondents. The Mann-Whitney test was applied for the analysis of quantitative variables. The scale data were analyzed using SPSS version 23 (IBM Corp), and the MNL model and LCM were adopted in Lighthouse Studio (version 9.9.1).

### Subgroup Analysis Procedure

The LCM was robust in identifying unobserved heterogeneity within the data, and this method allowed for the identification of latent classes of individuals who exhibited similar preferences or characteristics. However, the LCM was not appropriate for investigating the association between pandemic fatigue and preference heterogeneities, as covariates, such as sex, age, and mental health status, were not controlled. Therefore, to further explore the preference heterogeneities among the respondents on controlling sex, age, and mental health status, we also conducted subgroup analyses based on respondents’ demographic information, including sex, age, monthly income, and mental health status. Moreover, we conducted a subgroup analysis based on respondents’ levels (high level or low level) of pandemic fatigue according to the results of the pandemic fatigue scale.

### Ethical Considerations

We collected consent from respondents through an online consent form in the survey. This study has been approved by the Institutional Review Board (IRB) at the City University of Hong Kong (reference number: 11-2022-65-E). We adhered to the International Society for Pharmacoeconomics and Outcome Research (ISPOR) reporting guidelines for designing and reporting the research questions, assessing attributes and levels, and performing statistical analysis for the DCE.

## Results

### Respondent Characteristics

A total of 1183 respondents clicked on the link of our survey, and of these, 855 completed the survey. After the control process for data quality involving the exclusion of respondents who wrongly answered a trap question, 689 respondents were included in the final analysis. Among the 689 respondents, 341 (49.5%) were male and 348 (50.5%) were female. Additionally, 286 (41.5%) respondents were aged 35 years or younger. Most respondents (509/689, 73.9%) had a monthly income equal to or less than RMB 10,000 (US $1378.15), and 30.5% (210/689) of respondents were migrants (Table 2).

**Table 2:** Demographic and socioeconomic information of the respondents.

Characteristic		Value (N=689), n (%)
**Sex**		
	Male	341 (49.5)
	Female	348 (50.5)
**Age (years)**		
	18-25	128 (18.6)
	26-35	159 (22.9)
	36-45	135 (19.6)
	46-55	129 (18.7)
	≥56	139 (20.2)
**Education level**		
	Below bachelor’s degree	181 (26.3)
	Bachelor’s degree	401 (58.2)
	Above bachelor’s degree	107 (15.6)
**Current residence**		
	Northeast China	28 (4.1)
	North China	122 (17.7)
	East China	244 (35.4)
	Central China	105 (15.2)
	South China	103 (14.9)
	Southwest China	66 (9.6)
	Northwest China	21 (3.1)
**Original residence**		
	Northeast China	29 (4.2)
	North China	108 (15.7)
	East China	233 (33.8)
	Central China	143 (20.8)
	South China	86 (12.5)
	Southwest China	70 (10.2)
	Northwest China	20 (2.9)
**Religion**		
	Christianity	16 (2.3)
	Mohammedanism	3 (0.4)
	Buddhism	63 (9.1)
	Others	2 (0.3)
	None	605 (87.8)
**Marital status**		
	Unmarried and single	188 (27.3)
	Unmarried and cohabiting	21 (3.0)
	Married	469 (68.1)
	Divorced	9 (1.3)
	Widow	2 (0.3)
**Migrant**		
	Yes	210 (30.5)
	No	479 (69.5)
**Occupation and working status**		
	Student	130 (18.9)
	Manager	95 (13.8)
	Technician and associate professional	124 (18.0)
	Clerical support worker	105 (15.2)
	Service and sales worker	106 (15.4)
	Skilled agricultural, forestry, and ﬁshery worker	28 (4.1)
	Plant and machine operator and assembler	50 (7.3)
	Others	51 (7.4)
**Monthly income (RMB^a^)**		
	10,000 or below	509 (73.8)
	10,001 or above	180 (26.2)
**History of mental health disease**		
	Yes	42 (6.1)
	No	634 (92.0)
	Prefer not to say	13 (1.9)

^a^A currency exchange rate of 1 RMB=0.138 USD is applicable.

### Preferences for Public Health Measures

**Figure 2:**
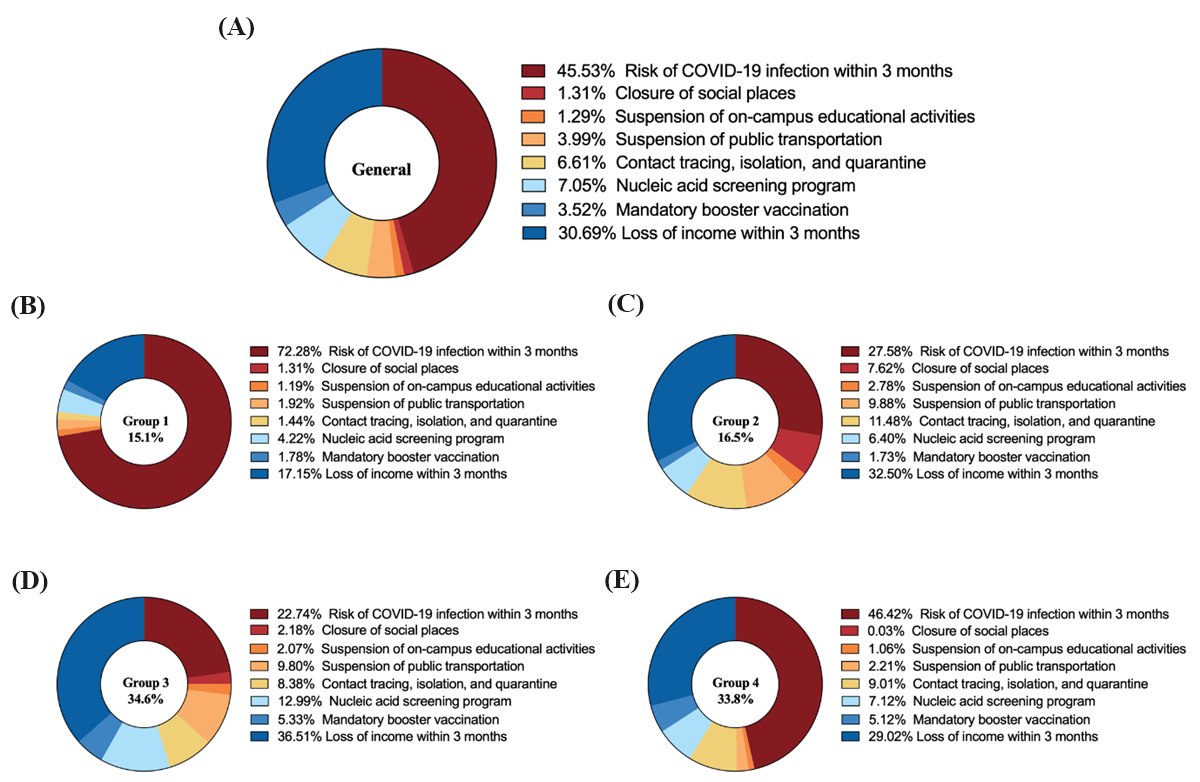
Weighted attribute importance among public and different latent classes of respondents. (A) Weighted attribute importance among all respondents. (B-E) Weighted attribute importance among 4 latent classes of respondents (groups 1-4). A larger proportion represents a higher attribute importance.

The attribute that had the most weighted importance in respondents’ decision-making was the risk of being infected with COVID-19 in 3 months (45.53%), followed by the loss of income due to COVID-19 measure (30.69%). Suspension of on-campus educational activities (1.29%) had the weakest weighted preference (Figure 2). Weaker preferences were observed when increasing the risk of infection with COVID-19. Compared with the full suspension of public transportation, respondents believed that suspension in only high-risk areas (areas with 10 or more local conﬁrmed cases were designated as high-risk areas) would be associated with larger utility (OR 1.168, 95% CI 1.106-1.234; *P*=.002). Moreover, compulsory contact tracing was favored by respondents compared with voluntary contact tracing (OR 1.294, 95% CI 1.225-1.366; *P*<.001). In addition, respondents were willing to accept booster doses of COVID-19 vaccines, and their utility decreased along with loss of income within 3 months due to PHSMs (Table 3).

**Table 3:** Respondents’ preferences and utilities of diﬀerent attribute levels.

Variable			Coefficient^a^		SE		*P* value		OR^b^ (95% CI)
**Risk of COVID-19 infection within 3 months**									
	0% (reference)	0.832		0.044		<.001		—^c^	
	20%	0.516		0.044		<.001		0.729 (0.669-0.795)	
	40%	0.299		0.044		<.001		0.587 (0.538-0.639)	
	60%	–0.171		0.046		<.001		0.367 (0.335-0.401)	
	80%	–0.535		0.049		<.001		0.255 (0.231-0.281)	
	100%	–0.941		0.055		<.001		0.170 (0.152-0.189)	
**Closure of social occasions**									
	Yes (reference)	–0.025		0.018		.16		—	
	No	0.025		0.018		.16		1.052 (1.016-1.090)	
**Suspension of on-campus educational activities**									
	Yes (reference)	–0.025		0.018		.16		—	
	No	0.025		0.018		.16		1.051 (1.015-1.089)	
**Suspension of public transportation**									
	Full suspension (reference)	–0.067		0.028		.02		—	
	Suspension in high-risk areas	0.088		0.028		.002		1.168 (1.106-1.234)	
	Normal operation	–0.021		0.028		.46		1.048 (0.992-1.106)	
**Contact tracing, isolation, and quarantine**									
	Voluntary (reference)	–0.098		0.028		<.001		—	
	Compulsory	0.159		0.028		<.001		1.294 (1.225-1.366)	
	None	–0.061		0.028		.03		1.038 (0.983-1.097)	
**Nucleic acid screening program**									
	Only high-risk units, workplaces, and vulnerable public (reference)	0.083		0.028		.003		—	
	Nucleic acid screening for all staff	0.096		0.028		<.001		1.013 (0.959-1.070)	
	None	–0.179		0.029		<.001		0.770 (0.728-0.814)	
**Mandatory booster vaccination**									
	Universal vaccination (reference)	0.082		0.028		.003		—	
	Only high-risk groups are vaccinated (long-term patients, people over 60 years old, etc)	–0.055		0.028		.047		0.872 (0.826-0.921)	
	None	–0.026		0.028		.35		0.898 (0.850-0.949)	
**Loss of income in 3 months**									
	0% (reference)	0.541		0.044		<.001		—	
	20%	0.426		0.044		<.001		0.891 (0.817-0.972)	
	40%	0.207		0.045		<.001		0.716 (0.656-0.782)	
	60%	–0.148		0.047		.002		0.564 (0.514-0.617)	
	80%	–0.371		0.048		<.001		0.561 (0.510-0.616)	
	100%	–0.655		0.051		<.001		0.302 (0.274-0.334)	

^a^The results were calculated using the multinomial logit model. A positive sign represents a positive utility for respondents choosing the specific level, and a negative sign represents a negative utility for respondents choosing the specific level.

^b^OR: odds ratio.

^c^Not applicable.

### Subgroup Analysis of Preferences for PHSMs

**Figure 3:**
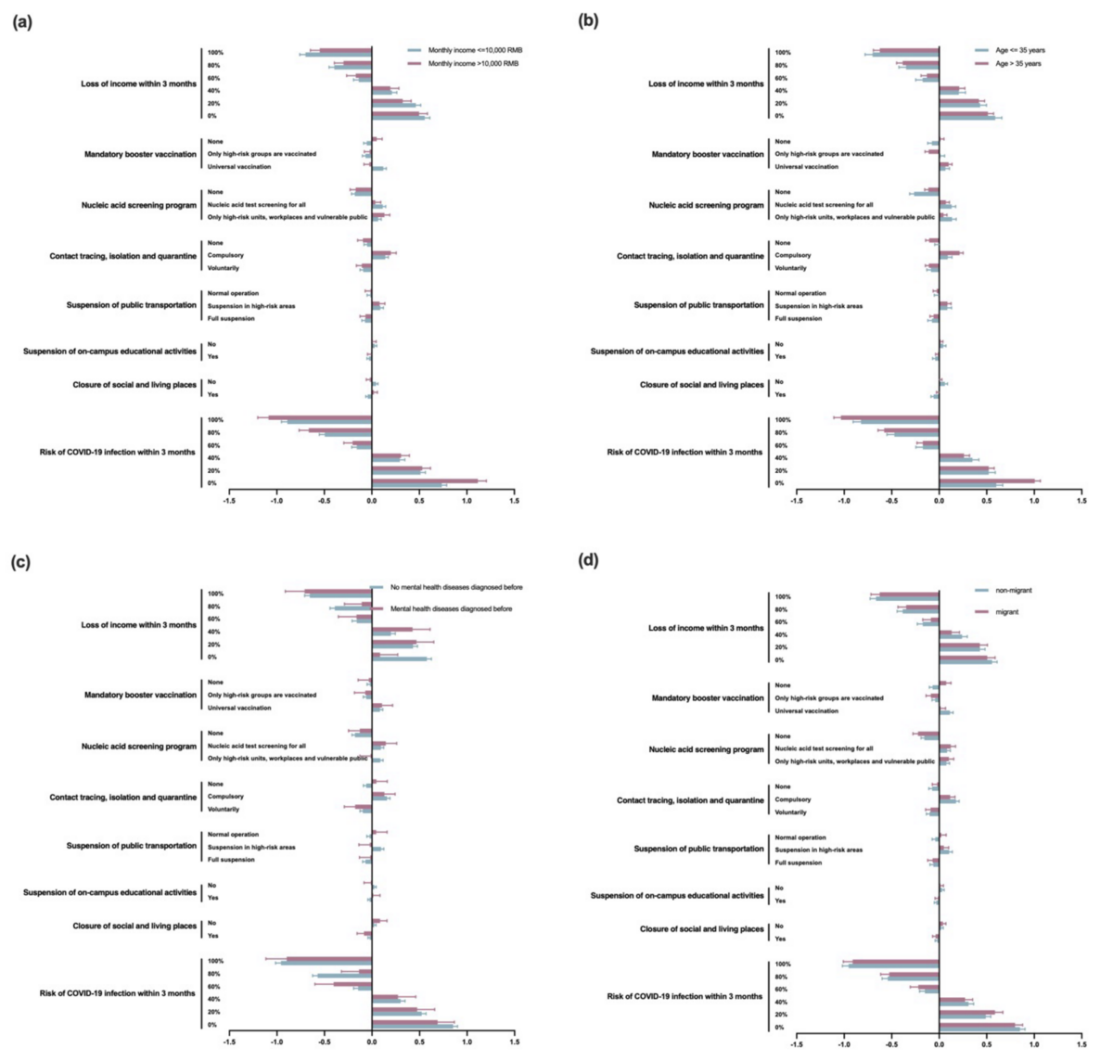
Subgroup analysis based on sex, age, mental health disease history, and residence status. (A) Subgroup analysis of preferences of respondents with a monthly income of ≤10,000 or >10,000 RMB. A currency exchange rate of 1 RMB=0.138 USD is applicable. (B) Subgroup analysis of preferences of respondents aged ≤35 years or >35 years. (C) Subgroup analysis of preferences of respondents with or without mental health diseases diagnosed previously. (D) Subgroup analysis of preferences of nonmigrant or migrant respondents.

To better trace the heterogeneities of the preferences, subgroup analyses were conducted in terms of age and diﬀerent monthly income levels (Figure 3). Compared with respondents having high monthly income, those having low monthly income were less sensitive to the risk of infection with COVID-19 within 3 months but more sensitive to the loss of income due to the measure within 3 months. Moreover, low-income respondents cared more about nucleic acid test screening for all and preferred the suspension of public transportation in only high-risk areas. Additionally, low-income respondents preferred not to be close to social and living places, which was in contrast with the findings for high-income respondents.

Similarly, respondents older than 35 years were more sensitive to the risk of COVID-19 infection and less sensitive to the loss of income within 3 months. Moreover, compared with older respondents, younger respondents preferred nucleic acid test screening for only high-risk units, workplaces, and vulnerable public, while older respondents preferred screening for all. Furthermore, younger respondents preferred not suspending on-campus educational activities and not closing social and living places.

Respondents diagnosed with mental health diseases did not favor contact tracing, isolation, and quarantine, as well as closure of social and living places compared with those without mental health diseases. The subgroup analysis for migrants and nonmigrants indicated that migrants had less acceptance of the mandatory booster vaccination and accepted the suspension of transportation in high-risk areas or normal operations.

### Subgroup Analysis of Pandemic Fatigue and Preference Heterogeneities

**Figure 4:**
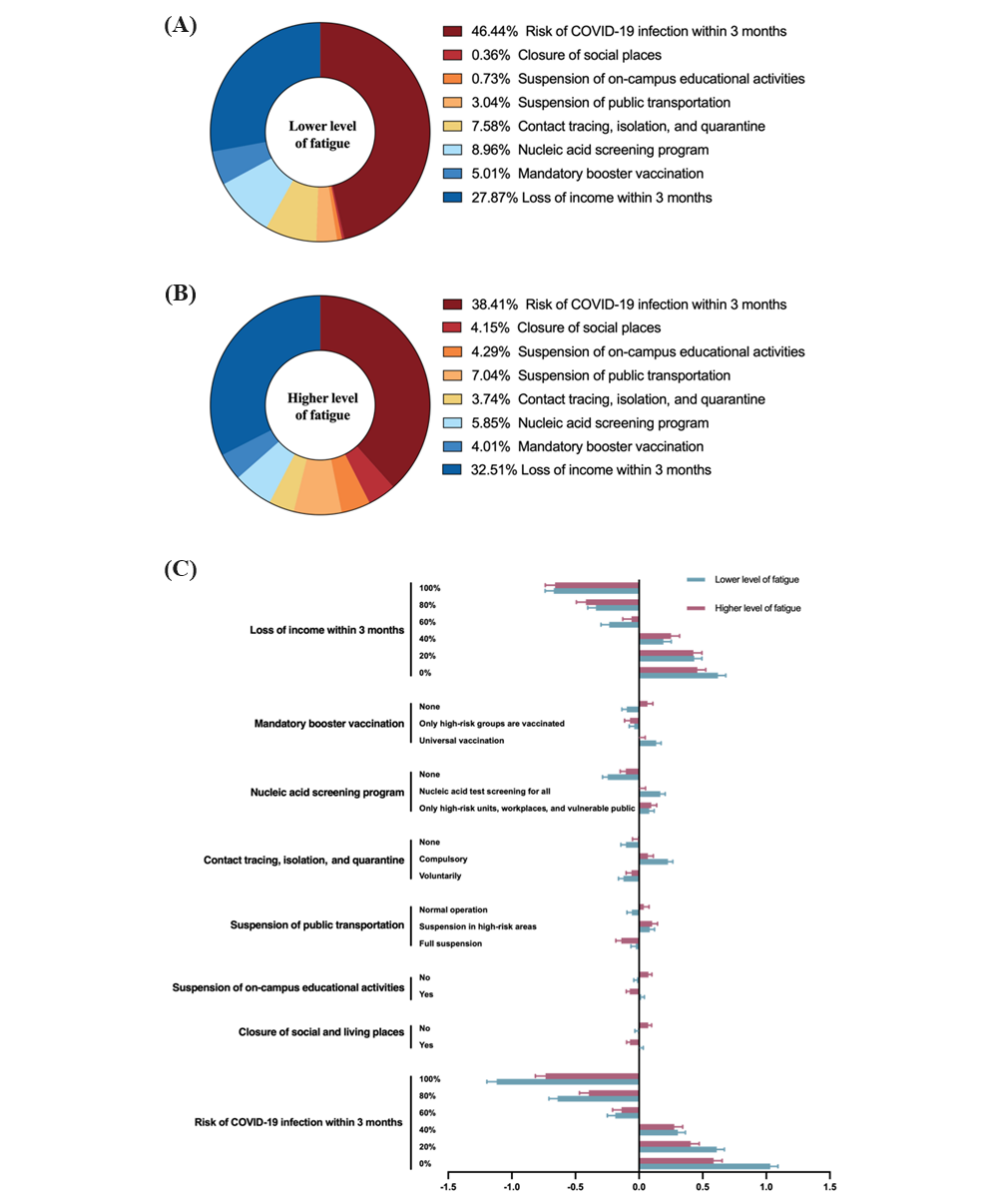
Weighted importance of attributes and levels among respondents based on pandemic fatigue levels. (A) Weighted attribute importance among respondents with a lower level of COVID-19 pandemic fatigue. (B) Weighted attribute importance among respondents with a higher level of COVID-19 pandemic fatigue. (C) Relative utility of levels among the 2 groups of respondents.

A higher pandemic fatigue level was observed in female respondents, younger respondents, migrants, and relatively lower-income respondents (COVID-19 Pandemic Fatigue Scale [CPFS] correlation with age: *r*=–0.274, *P*<.001; correlation with monthly income: *r*=–0.25, *P*<.001) (Table 4). Based on the results of the CPFS, some preference heterogeneities were also found among respondents with a lower or higher level of pandemic fatigue (Figure 4). Respondents with a higher level of fatigue tended to be less sensitive to the risk of COVID-19 infection within 3 months and more sensitive to income loss within 3 months. Additionally, compared with respondents with a lower level of pandemic fatigue, those with a higher level of fatigue preferred the nonsuspension of social places and nonsuspension of on-campus educational activities. Mandatory booster COVID-19 vaccination was also not preferred by respondents with a higher level of pandemic fatigue, while universal COVID-19 booster vaccination was preferred by respondents with a lower level of pandemic fatigue.

**Table 4:** Results of the COVID-19 Pandemic Fatigue Scale.

Variable			Respondents, n		Score, mean (SD)		*P* value^a^		Correlation^b^		*P* value
All respondents			689		15.24 (5.262)				—^c^		—
**Gender**							.01		—		—
	Male	341		14.71 (5.350)							
	Female	348		15.75 (5.120)							
**Age (years)^d^**							<.001		–0.274		<.001
	18-25	128		18.28 (4.836)							
	26-35	158		15.01 (4.939)							
	36-45	135		14.50 (4.982)							
	46-55	129		15.08 (5.758)							
	≥56	139		13.55 (4.671)							
**Education level**							.18		—		—
	Middle school education or below	26		16.54 (5.798)							
	High school education	63		14.87 (3.744)							
	Vocational school education	92		14.63 (5.353)							
	Bachelor’s degree	401		15.07 (5.214)							
	Master’s degree	97		16.12 (5.938)							
	PhD degree	10		17.50 (5.255)							
**Religion**							.29		—		—
	Christianity	16		13.19 (4.490)							
	Mohammedanism	3		18.33 (7.506)							
	Buddhism	63		14.89 (5.873)							
	Others	2		17.00 (6.272)							
	None	605		15.30 (5.197)							
**Marital status**							<.001		—		—
	Unmarried and single	188		17.65 (4.989)							
	Unmarried and cohabiting	21		16.81 (4.633)							
	Married	469		14.08 (4.979)							
	Divorced	9		20.44 (5.175)							
	Widow	2		18.50 (10.607)							
**Migrant**							.001		—		—
	Yes	210		16.12 (5.288)							
	No	479		14.85 (5.209)							
**Occupation and working area**							<.001		—		—
	Student	130		18.58 (4.767)							
	Manager	95		14.18 (5.357)							
	Technician and associate professional	124		14.42 (5.516)							
	Clerical support worker	105		13.65 (4.218)							
	Service and sales worker	106		15.26 (5.231)							
	Skilled agricultural, forestry, and fishery worker	28		16.54 (56.215)							
	Plant and machine operator and assembler	50		13.10 (3.754)							
	Others	51		15.25 (4.560)							
**Monthly income (RMB^e^)^d^**							<.001		–0.25		<.001
	≤5000	220		16.76 (4.985)							
	5000-10,000	289		14.78 (4.768)							
	10,001-15,000	103		14.45 (5.656)							
	15,001-20,000	47		13.45 (6.064)							
	≥20,000	30		14.00 (6.623)							
**History of mental health disease**							.02		—		—
	Yes	42		16.95 (5.635)							
	No	634		15.04 (5.194)							
**Exposure to novel coronavirus pneumonia**							.14		—		—
	Yes	198		15.84 (5.613)							
	No	491		14.99 (5.099)							
**Closed-off community management^f^**							.42		—		—
	Yes	90		15.93 (6.005)							
	No	599		15.13 (5.138)							

^a^Mann-Whitney test.

^b^Spearman correlation coefficients for noncontinuous variables.

^c^Not applicable.

^d^Continuous variable.

^e^A currency exchange rate of 1 RMB=0.138 USD is applicable.

^f^Entrance numbers were minimized, checking points were set up in communities, entry permits were limited, face mask wearing was required, health monitoring was enhanced, and only registered personnel and vehicles were allowed to pass through.

### Latent Class Analysis

According to the AIC and BIC of the LCM, 4 latent groups of respondents were determined, with the lowest BIC value of 9680.29 and AIC value of 9104.82. All the other model fitting values have been presented in Multimedia Appendix 3. The segmented sizes were 15.1%, 16.5%, 34.6%, and 33.8% for groups 1, 2, 3, and 4, respectively. As shown in Figure 2, respondents in groups 1 and 4 attached the most importance to the risk of COVID-19 infection within 3 months, while respondents in groups 2 and 3 attached importance to the loss of income within 3 months. Additionally, following the risk of COVID-19 infection and loss of income within 3 months, groups 2 and 4 considered contact tracing and nucleic acid test screening to be the third and fourth most important attributes, respectively. Group 3 believed that nucleic acid test screening and suspension of public transportation were very essential.

## Discussion

The COVID-19 pandemic posed tremendous challenges for delivering mental and physiological health services throughout China. This study sought to comprehensively investigate public mental health and preferences for PHSMs. This is the first study to estimate public preferences for PHSMs using a DCE for a nationally representative population in China. The risk of COVID-19 infection within 3 months; contact tracing, isolation, and quarantine; nucleic acid screening program; and loss of income within 3 months significantly influenced the preferences for PHSMs.

In our study, we found that the respondents placed the greatest importance on the risk of COVID-19 infection in the last 3 months when considering public health measures for COVID-19 mitigation. With its rapid spread and serious complications, COVID-19 caused fear in the vast majority of people irrespective of whether they were in the risk group. In a population-based survey conducted in America [45], the population was fearful, worried, and uncertain about COVID-19, especially in more densely populated communities, communities with higher presumptive and reported COVID-19 case concentrations, and urban locations. Additionally, an online survey in Italy that asked about health behaviors and the psychological and overall impact of COVID-19 found that only the fear of infection significantly dissuaded people from violating epidemic prevention rules [46]. Hence, the risks of infection and adverse outcomes secondary to infection should be clearly outlined by the media or the government to the public to enhance mutual understanding, reduce their psychological burden, and improve the compliance of people’s epidemic prevention behavior.

Furthermore, the respondents in our survey attached more importance to income loss in their preferences. According to an analysis based on economic forecasts in the European Union, the COVID-19 crisis had an indispensable impact on household disposable income, similar to the one experienced during the 2008-2009 financial crisis, with lower-income households being more severely hit [47]. The high preference may be due to the large negative economic, living, and psychological effects of lower income [48]. This was consistent with the fact that migrant workers, accounting for about one-fifth of the whole Chinese population, were faced with large housing stress and psychological burden from the sudden loss of income and further quarantine enforcement during the COVID-19 pandemic [31]. Therefore, the government should consider subsidies related to epidemic prevention, particularly for the low-income population; take fiscal policy measures as appropriate to reduce the risk and scale of income reduction; and cushion the impact of the epidemic crisis on inequality and poverty through policy interventions.

Respondents in our study showed preference heterogeneity for epidemic prevention measures. Understanding the heterogeneity of information and differences in personal values toward epidemic prevention measures can help policy makers understand individuals’ preferences so that more rational and customized PHSMs can be formulated to reduce the negative emotions caused by epidemic prevention. For example, younger participants preferred not to undergo nucleic acid screening, but older people were more afraid of having novel coronavirus pneumonia. The probable cause is that the case fatality rate of novel coronavirus pneumonia is low in young people and increases in a log-linear model by age among individuals older than 30 years [49]. Therefore, relevant departments should be responsible for community humanistic care, appeasing the mood of the masses, eliminating panic, guiding the community to carry out scientific and orderly epidemic prevention work, implementing vaccine booster shots in the population, and publicizing the scientific knowledge of COVID-19.

Our study showed that migrant workers had a high level of pandemic fatigue related to the suspension of transportation and closure of social places, which aligned with existing literature [31,50]. These findings indicate that vulnerable groups, including migrants and the older population [51], are more prone to experience psychological pressure due to unemployment, suspension of the public transportation network, and loss of income [12,52]. These findings emphasize the importance of psychological placation for susceptible populations during the outbreak to help provide support and managed care for individuals at risk of psychological impact. On the other hand, we found higher pandemic fatigue scores in young participants than in participants from other age groups, which is consistent with the finding in a previous study [53] reporting that university students had significantly reduced mood and reduced social interactions during lockdown periods. For migrant workers, elderly people, and other susceptible populations, governments should develop effective mental health interventions and strategies, carefully assess and manage the mental health needs of vulnerable groups, and provide mental health services through community management or digital platforms during the epidemic.

Although COVID-19 PHSMs are dynamic, our findings contributed to the existing literature by providing a better understanding of the psychological impact of the pandemic, and this may be useful for formulating and planning effective prevention strategies and psychological counseling for the public and susceptible populations. Moreover, the findings of this study may provide insights for PHSM design when managing epidemic outbreaks in the future. Through the analysis of heterogeneous populations that have been affected by the pandemic mentally and emotionally, our research provides key insights that can inform formulation and priority settings and the planning of more effective prevention strategies and psychological support mechanisms. This is particularly relevant for public health authorities and policy makers who are challenged and tasked with conditions involving the physical and mental well-being of the public and vulnerable groups during such crises. Furthermore, the implications of our findings extend far beyond the current pandemic context. As we investigated the psychological effects of COVID-19 and the preferences of various PHSMs for mitigating these impacts, we were able to provide insights that can be pivotal in the face of future infectious disease outbreaks. Moreover, our research highlights the necessity of incorporating psychological considerations into the priority settings of PHSMs. This approach ensures that interventions are holistic, addressing both the epidemiological and emotional aspects of disease control.

There are limitations in this study, especially in the sampling methods. As we applied quota sampling without providing the quotas of regions in this study owing to budget issues, the results may have a potential bias for inferring the general population, and selection bias may also exist. Moreover, in our study, we collected preference data from 689 respondents living in 31 provinces in China. However, considering the 1.4 billion population in China, the presence of only around 22 respondents in each province may reduce the representativeness of the sampling. Owing to the limited budget for data collection and the restricted offline sampling procedure related to the COVID-19 pandemic lockdown, the flexibility of the sampling was largely limited. Further studies with larger and more representative samples for investigating the mental health of the general population under the conditions of the pandemic may be required to more accurately quantify the perspectives for PHSMs. In addition, the study acknowledges the limitations imposed by the use of quota sampling, particularly the equal representation of sexes and the simplified categorization of age groups, which may not accurately reflect the complex demographics of the adult population in China. Owing to the challenges posed by the pandemic and the budgetary constraints for data collection, the study could not completely adhere to the exact adult age structure of the Chinese population in the sampling methods. This limitation may affect the generalizability of the findings to the entire adult population of China. This is a limitation that future research might overcome with alternative strategies or under different circumstances. Finally, we acknowledge that the DCE questionnaire may impose some cognitive burden on respondents, and this may lead to some biases when selecting among the alternative options. Therefore, a face-to-face approach is considered to be better than an online approach. However, due to the pandemic lockdown, a face-to-face approach was not feasible. In future research performed to understand people’s pandemic fatigue and preferences, a face-to-face approach should be applied if there is no lockdown.

Variability in the preference for COVID-19 policies was found between different groups. Pandemic fatigue and fear of COVID-19 infection contributed to the public’s mental health problems. Hence, at the late stage of the pandemic, policy makers should consider reducing people’s mental burden by introducing approaches to relieve people’s fear of infection when PHSMs are being relaxed. The findings provide insights on PHSM implementation for outbreaks in the future as our research highlights the necessity of incorporating heterogeneous psychological considerations into the priority settings of PHSMs. This may ensure that interventions are holistic, addressing both the epidemiological and emotional aspects of disease control.

## Appendix

Multimedia Appendix 1 Study survey.

Multimedia Appendix 2 Sample size calculation (standard parametric approach).

Multimedia Appendix 3 Akaike Information Criterion, Bayesian Information Criterion, and other fitting values.

## Abbreviations

AIC: Akaike Information Criterion
BIC: Bayesian Information Criterion
CPFS: COVID-19 Pandemic Fatigue Scale
DCE: discrete choice experiment
LCM: latent class model
MNL: multinomial logit
OR: odds ratio
PHSM: public health and social measure

## Data Availability

The data related to this study can be obtained upon reasonable request to the corresponding author.
