# Supplementary material for: Pandemic Fatigue and Preferences for COVID-19 Public Health and Social Measures in China: Nationwide Discrete Choice Experiment
Source: JMIR Public Health Surveill. 2024 Jun 27;10:e45840. doi: 10.2196/45840 (PMC11240073; doi:10.2196/45840)
Supplement: Multimedia Appendix 1 [file publichealth_v10i1e45840_app1.pdf]

Multimedia Appendix 1. Study survey.

Hello! Thank you very much for participating in our survey!

All data in this survey will be collected anonymously and will be kept strictly confidential. This questionnaire will consist of four parts and will take about 10 minutes to complete. Do you agree to participate?

- Agree
- Do not agree to participate in the survey

Are you over 18 years old?

- Yes
- No

This survey is anonymous, and we promise to keep your information confidential, the survey results will not be used for commercial purposes. Please feel free to answer according to your own situation. If you have any questions, you are welcome to contact us via email. Email: 2752209735@qq.com

Part I Demographic Information In this section, we will ask you questions about basic information, so please answer truthfully.

1. What is your sex?
   - Male
   - Female

2. What is your age?
   - 18-25 years old
   - 26-35 years old
   - 36-45 years old

- 46-55 years old

  56-65 years old

- 65 years old or more

3. What is your highest education level?
   - Junior high school and below
   - High school
   - Specialized education (or other equivalent)
   - Undergraduate
   - Master's degree
   - Doctoral degree and above

4. What is your religion?
   - Christianity
   - Islam
   - Buddhism
   - Other, please specify
   - None

5. What is your marital status?
   - Married
   - Single
   - Unmarried
   - Cohabiting
   - Married
   - Divorced
   - Widowed

6. Please select the province and city where you are currently living.

7. How many years have you stayed in your current place of residence?

-

Less than 1 year

- 1~5 years
- 6~10 years
- 11~15 years
- 16~20 years
- More than 20 years.

8. Please select the province and city of your household registration.

9. Which industry do you work in?
   - Head of state organs, party organizations, enterprises and institutions
   - Professional and technical personnel (doctors, lawyers, architects, etc.)
   - Clerks and related personnel (clerks)
   - Commercial and service personnel
   - Agricultural, forestry, animal husbandry, fishery and water conservancy personnel
   - Operators of production and transportation equipment and related personnel
   - Military personnel Students
   - Others, please specify

10. What is your average monthly income?
    - Less than 5,000 RMB • 5,000~10,000 RMB
    - 10,001~15,000 RMB
    - 15,001-20,000 RMB
    - 20,000 RMB or more

11. What is the type of vaccine you are currently receiving? (Multiple choices allowed)
    - Inactivated vaccine (e.g. Beijing Biotechnology, Wuhan Biotechnology, Sinovac)
    - Messenger RNA vaccine (e.g. Comirnaty)
    - Adenovirus vector vaccine (e.g. Kangxinuo)

    Recombinant subunit vaccine (e.g. Zhifeilongkoma)

12. Do you have a history of diagnosed mental health disorders (e.g., depression, anxiety, obsessive-compulsive disorder, phobias, bipolar disorder, neurodegeneration, schizophrenia, personality disorder, etc.)?

- Yes
- No
- I don't want to say

13. Have you ever been diagnosed with or are you currently suffering from any of the following mental illnesses?

- Depression
- Anxiety
- Obsessive-Compulsive Disorder
- Phobia
- Bipolar Disorder
- Neurasthenia
- Schizophrenia
- Personality Disorder
- Other, please describe.

14. What is the severity of your current or former mental illness?

- Not severe
- Moderate
- Very severe

15 Have you ever received treatment for a mental health disorder?

- Yes
- No

•

Part II Health Status Questionnaire Related Surveys

This section contains a number of questionnaires on mental health, the main purpose of which is to explore the emotional well-being of the population in the context of the national response to the epidemic.

1. In the past 2 weeks, how often have you been bothered by the following? (from not at all, a few days, more than half of the days, almost every day)
2. During the past two weeks, have you been regularly troubled by the following problems? (from not at all, a few days, more than seven days, almost every day)
3. If you have any of these problems, how much difficulty do they cause you at work, at home, or with people? (not at all, somehow, very difficult, extremely difficult)

Part III Public Preferences for Public Health Socialization Measures in a New Crown Epidemic

A Discrete Choice Experiment (DCE) is a research method used to measure preferences, values, and choices among different alternatives. It involves presenting respondents with a series of choices between sets of alternatives, where each alternative is described by a set of attributes. The responses are used to analyze the trade-offs people are willing to make between different attributes, helping researchers understand the relative importance of these attributes in decisionmaking. DCEs are widely used in health economics, marketing, environmental economics, and transport planning to inform policy-making and product design.

In this part, you will face a series of tasks; these are called discrete choice tasks, a method we use to understand preferences and decision-making processes. Each task will offer you two hypothetical options and a “none” option, each with a set of attributes or features. Your task is to choose the option that you prefer or that you would most likely choose in real life. Please read the descriptions of each option carefully. Each option is different, with its unique set of attributes or characteristics. Remember, there's no right or wrong answer here. We are interested in your genuine preferences. Choose the option that best aligns with what you would prefer in real life, based on the attributes presented. Some scenarios may not happen in real life since they were hypothetical; however, please also select an alternative based on your own preferences.

A sample of DCE:

Assuming that the Government will now implement two different sets of public health measures to interrupt the spread of the COVID-19, please select your preferred option among the following two hypothetical public health measures.

Assuming that the government will implement two different sets of public health measures to mitigate the spread of the coronavirus, please select one of the following two hypothetical public health measures that you prefer

(Q1, 9 questions in total) Please note: some scenarios may not be realistic, but please also choose the one that better suits your preference based on the information given

	**Option 1**	**Option 2**	**None of both**
Risk of COVID-19 infection within 3 months	60%	100%	
Closure of social occasion	Yes	No	
Suspension of on-campus educational activities	Yes	No	
Suspension of public transportation	Suspension in high-risk areas	Normal operation	None of both
Contact tracing, isolation and quarantine	Voluntarily	None	
Nucleic acid screening program	Only high-risk units, workplaces and vulnerable public	None	
Mandatory booster vaccination	None	Universal vaccination	
Loss of income in 3 months	100%	40%	
	select	select	select

Next Page

Part IV. Attitude or opinion of the public towards the strengthening of measures for epidemic prevention and control at this stage

This part of the questionnaire will be used to explore people's attitudes or views on the strengthening of measures related to the prevention and control of the epidemic at this stage, please choose according to your actual situation.

1. Are there any locally confirmed cases in your area (town, county, district)?
2. What are the current public health social measures in your area?
3. Please rate your opinion on the following 12 statements. (from totally disagree to totally agree)
    - I am highly susceptible to the SARS-CoV-2.
    - I will have severe sequelae if infected with the SARS-CoV-2.
    - The current variant of (Omicron variant) leads to a higher mortality rate.
    - With current immunization measures, my health condition is more plagued by chronic illnesses compared to infection with COVID-19.
    - With current immunization measures, I have an increased risk of death from COVID-19
    - I am unable to take proper personal protection because of shortage of immunization materials (masks/sterilizers, etc.)
    - I am unable to take proper personal protection because of the high cost of epidemic prevention materials (masks/sanitizers, etc.).
    - Compliance with current immunization measures protects me from COVID-19 infections.
    - Adhering to current immunization measures will protect my family from COVID-19.
    - I will strictly follow the public health social measures.
    - Television and news reports can encourage me to comply with current public health social measures.

- Fear of getting COVID-19 makes me comply with current public health social measures.

4. Please rate your knowledge, attitudes, and practices regarding the current epidemic prevention measures as probed by the following 10 statements. (from totally disagree to totally agree)
    - I know a lot about the current epidemic strain.
    - I have a good understanding of the clinical symptoms of COVID-19.
    - I am well-informed about the current public health social measures implemented in my area.
    - I am satisfied with the current public health social measures implemented in my local area.
    - I think it is important to adhere to the "Dynamic Zeroing" measures (Dynamic Zeroing refers to the current situation when there is a local case of the disease, through the adoption of comprehensive measures to achieve rapid eradication of the epidemic.)
    - I think the measure of "living with the epidemic" is very important (referring to the future use of vaccines to reduce the death rate and the spread of diseases, and to raise the awareness of the population in preventing and controlling the disease, so as to achieve peaceful coexistence with the virus)
    - I follow the vaccination recommendations of the immunization public health social measures.
    - I actively comply with vaccination requirements.
    - I strictly follow the requirements of the public health social measures "for personal protection".
    - I pay close attention to news reports related to public health social measures.

5. Please rate your fatigue with the current increased immunization measures by the following 6 statements.
    - I am tired of following the news about the COVID-19 outbreak.

- I don't want to try to comply with the changing public health social measures anymore.
- I am tired of the outbreak.
- I am tired of restraining myself to avoid infection from susceptible people.
- I try to change the subject when people around me talk about the COVID-19 because I don't want to talk about it.
- I feel like I am losing my will to fight the pandemic.
