# Supplementary material for: Pandemic Fatigue and Preferences for COVID-19 Public Health and Social Measures in China: Nationwide Discrete Choice Experiment
Source: JMIR Public Health Surveill. 2024 Jun 27;10:e45840. doi: 10.2196/45840 (PMC11240073; doi:10.2196/45840)
Supplement: Multimedia Appendix 2 [file publichealth_v10i1e45840_app2.pdf]

Multimedia Appendix 2. Sample size calculation (standard parametric approach).

We relied on the standard parametric approach for a choice probability to estimate the minimum sample size of this DCE study based on the equation below:

$$n \geq \frac{(1-p)}{rpa^2} \times \left(\phi^{-1}\left(1-\frac{\alpha}{2}\right)\right)^2$$

Where the p indicates the true population probability, in this study, we assume that 80% of the population adheres to the PHSMs, therefore, p was 0.8 in our study. r indicates the number of choice tasks per respondent; in our study, there are 9 random choice tasks. a indicates the accuracy level around the population probability, in our study, a was assumed to be 0.4. ϕ^{-1} indicates the inverse of the cumulative normal distribution function and α indicates the significance level 95%.
