# Supplementary material for: Pandemic Fatigue and Preferences for COVID-19 Public Health and Social Measures in China: Nationwide Discrete Choice Experiment
Source: JMIR Public Health Surveill. 2024 Jun 27;10:e45840. doi: 10.2196/45840 (PMC11240073; doi:10.2196/45840)
Supplement: Multimedia Appendix 3 [file publichealth_v10i1e45840_app3.pdf]

Multimedia Appendix 3. Akaike Information Criterion, Bayesian Information Criterion, and other fitting values.

Summary of best replications

Groups	Replication	Log-likelihood	Pct Cert	AIC	CAIC	BIC	ABIC	Chi-Square	Relative Chi-Square
2	2	-4682.61	22.67	9451.22	9778.65	9735.65	9599.01	2745.89	63.86
3	2	-4561.20	24.68	9252.41	9747.36	9682.36	9475.81	2988.70	45.98
4	3	-4465.41	26.26	9104.82	9767.29	9680.29	9403.83	3180.29	36.56
5	5	-4409.59	27.18	9037.18	9867.18	9758.18	9411.81	3291.93	30.20

Minimum number of groups	2
Maximum number of groups	5
Number of replications	5
Maximum number of iterations	100
Convergence limit for log-likelihood	0.00100
Standard errors reported	
Random number seed	1
Null log-likelihood	-6055.55

Data Summary

Iteration	Log-likelihood	Gain	Segment 1 Size	Segment 2 Size	Segment 3 Size	Segment 4 Size
0	-6055.55					
1	-5292.34	763.21	29.1	26.2	21.1	23.6
2	-4989.98	302.36	35.4	25.8	21.1	17.7
3	-4702.18	287.80	38.6	25.5	21.5	14.5
4	-4639.53	62.65	38.5	24.7	21.4	15.5
5	-4598.27	41.27	37.8	23.3	20.8	18.1
6	-4554.83	43.43	37.1	22.0	19.9	20.9
7	-4524.28	30.56	36.2	21.0	19.5	23.3
8	-4509.10	15.17	35.3	20.3	19.4	25.1
9	-4502.13	6.98	34.4	19.8	19.5	26.4
10	-4498.45	3.68	33.5	19.3	19.8	27.4
11	-4496.02	2.43	32.6	19.0	20.2	28.2
12	-4494.04	1.98	31.7	18.7	20.7	28.9
13	-4492.18	1.86	30.8	18.4	21.3	29.5
14	-4490.29	1.89	29.9	18.2	21.9	30.0
15	-4488.32	1.97	29.0	18.0	22.6	30.4
16	-4486.30	2.02	28.0	17.8	23.4	30.8
17	-4484.26	2.03	27.1	17.6	24.1	31.1
18	-4482.27	1.99	26.2	17.5	24.9	31.5
19	-4480.38	1.89	25.3	17.3	25.6	31.8
20	-4478.62	1.76	24.4	17.2	26.4	32.1
21	-4476.99	1.62	23.6	17.0	27.0	32.3
22	-4475.51	1.48	22.8	16.9	27.7	32.6
23	-4474.16	1.35	22.1	16.8	28.3	32.8
24	-4472.94	1.22	21.5	16.7	28.9	33.0
25	-4471.85	1.09	20.8	16.6	29.4	33.2
26	-4470.90	0.96	20.3	16.5	29.9	33.4
27	-4470.06	0.83	19.8	16.5	30.3	33.5
28	-4469.35	0.72	19.3	16.4	30.7	33.7
29	-4468.73	0.62	18.9	16.3	31.0	33.8
30	-4468.20	0.53	18.5	16.3	31.3	33.9
31	-4467.75	0.45	18.1	16.3	31.6	34.0
32	-4467.36	0.39	17.8	16.2	31.9	34.1

33	-4467.03	0.33	17.5	16.2	32.1	34.1
34	-4466.75	0.28	17.3	16.2	32.3	34.2
35	-4466.51	0.24	17.0	16.2	32.5	34.2
36	-4466.31	0.20	16.8	16.2	32.7	34.2
37	-4466.14	0.17	16.6	16.2	32.9	34.3
38	-4466.01	0.14	16.5	16.2	33.1	34.3
39	-4465.89	0.11	16.3	16.2	33.2	34.3
40	-4465.80	0.09	16.2	16.2	33.3	34.3
41	-4465.73	0.08	16.0	16.2	33.5	34.3
42	-4465.67	0.06	15.9	16.3	33.6	34.2
43	-4465.62	0.05	15.8	16.3	33.7	34.2
44	-4465.58	0.04	15.7	16.3	33.8	34.2
45	-4465.54	0.03	15.7	16.3	33.9	34.2
46	-4465.52	0.03	15.6	16.3	33.9	34.2
47	-4465.50	0.02	15.5	16.3	34.0	34.1
48	-4465.48	0.02	15.5	16.3	34.1	34.1
49	-4465.47	0.01	15.4	16.4	34.1	34.1
50	-4465.46	0.01	15.4	16.4	34.2	34.1
51	-4465.45	0.01	15.3	16.4	34.2	34.0
52	-4465.44	0.01	15.3	16.4	34.3	34.0
53	-4465.43	0.01	15.3	16.4	34.3	34.0
54	-4465.43	0.00	15.3	16.4	34.3	34.0
55	-4465.42	0.00	15.2	16.4	34.4	33.9
56	-4465.42	0.00	15.2	16.5	34.4	33.9
57	-4465.42	0.00	15.2	16.5	34.4	33.9
58	-4465.42	0.00	15.2	16.5	34.5	33.9
59	-4465.41	0.00	15.1	16.5	34.5	33.9
60	-4465.41	0.00	15.1	16.5	34.5	33.9
61	-4465.41	0.00	15.1	16.5	34.5	33.8
62	-4465.41	0.00	15.1	16.5	34.5	33.8
63	-4465.41	0.00	15.1	16.5	34.5	33.8
64	-4465.41	0.00	15.1	16.5	34.6	33.8

Percent Certainty	26.26
Akaike Info Criterion	9104.82

Consistent Akaike Info Criterion	9767.29
Bayesian Information Criterion	9680.29
Adjusted Bayesian Info Criterion	9403.83
Chi-Square	3180.29
Relative Chi-Square	36.56

The average maximum membership probability is 0.89218.
